# Single-cell transcriptomic analysis in two patients with rare systemic autoinflammatory diseases treated with anti-TNF therapy

**DOI:** 10.3389/fimmu.2023.1091336

**Published:** 2023-02-24

**Authors:** Yichao Hua, Na Wu, Junke Miao, Min Shen

**Affiliations:** Department of Rheumatology and Clinical Immunology, Chinese Academy of Medical Sciences and Peking Union Medical College, National Clinical Research Center for Dermatologic and Immunologic Diseases (NCRC-DID), Ministry of Science & Technology, State Key Laboratory of Complex Severe and Rare Diseases, Peking Union Medical College Hospital (PUMCH), Key Laboratory of Rheumatology and Clinical Immunology, Ministry of Education, Beijing, China

**Keywords:** systemic autoinflammatory disease, single-cell RNA sequencing, anti-TNF therapy, Etanercept, cryopyrin-associated periodic syndromes (CAPS), TNF receptor-associated periodic fever syndrome (TRAPS)

## Abstract

Systemic autoinflammatory diseases (SAIDs) are a group of rare diseases characterized by recurrent or continuous inflammation, typically accompanied by genetic variants. Good responses to anti-TNF therapy were observed in SAIDs patients. However, the mechanisms underlying the disease flare and the response to TNF blocking therapy have not been fully elucidated. Here, single-cell RNA sequencing technology was used to describe the transcriptomic profile of PBMCs and PMNs in two SAID patients both before and after anti-TNF treatment. Interferon responses were involved in the disease flare. After anti-TNF therapy, clinical symptoms were alleviated while TNF and IL-1 were unexpectedly increased, indicating that these inflammatory cytokines are not positively correlated with disease activity. Trajectory analysis showed that inhibition of macrophage differentiation, rather than reduction of the inflammatory cytokines, as the potential mechanism of anti-TNF treatment response in SAIDs.

## 1 Introduction

Systemic autoinflammatory diseases (SAIDs) are a group of rare diseases caused by defects or dysregulation of the innate immune system, characterized by recurrent or continuous inflammation (e.g. fever, serosal, synovial, or cutaneous inflammation) and by the lack of a primary pathogenic role of the adaptive immune system (autoreactive T-cells or autoantibodies production) (1–4). This group of diseases includes more than 40 monogenic, as well as several polygenic diseases (5, 6). Classic monogenic SAIDs include familial Mediterranean fever (FMF, caused by *MEFV* gene variants), TNF receptor-associated periodic fever syndrome (TRAPS, by autosomal dominant variants in the *TNFRSF1A* gene), cryopyrin-associated periodic syndromes (CAPS)/*NLRP3*-associated autoinflammatory disease (*NLRP3*-AID, by gain-of-function variants in the *NLRP3* gene), and hyper-IgD syndrome (HIDS)/mevalonate kinase deficiency (MKD, by autosomal recessive variants in the *MVK* gene) (6). Patients with high disease activity can present with recurrent attacks of fever and other inflammatory symptoms, and a successful treatment can reduce disease activity, as manifested by reduced frequency or severity of disease flare or no flare. Regarding to pathogenesis, these classic SAIDs are classified as IL-1 mediated SAIDs, as the variants can directly or indirectly promote the assembly of inflammasomes that proteolytically process the inactive pro-IL-1β into active IL-1β. This can occur by increased intracellular sensor/pattern-recognition receptors (PRR) function (e.g. CAPS, FMF), generation of intracellular stress (e.g. TRAPS, HIDS), or loss of a negative regulator (e.g. Deficiency of IL-1 receptor antagonist, DIRA) (6). A lot of studies focus on the pathogenic role of the inflammasome – IL-1 axis in these SAIDs, and many patients have good responses to IL-1-blocking agents (e.g. anakinra, canakinumab, and rilonacept), although some patients with FMF, TRAPS or HIDS are less responsive to IL-1 inhibition (7, 8; 9–15). Little is known about other pathways in the pathogenesis of SAIDs, and the treatment response to other biological agents, e.g. TNF or IL-6 blocking agents. Some studies have reported increased NFκB activation and defective autophagy as potential pathogenic mechanisms (16–18). In our previous study, we observed a good response to TNF inhibitors in CAPS patients (19). Whether there are other pathways involved in the pathogenesis of SAIDs, and what the underlying mechanisms are in response to TNF blocking therapy, remain to be elucidated.

Single-cell RNA sequencing (scRNAseq) is a powerful technique to detect transcriptomics of single cells, which can further reveal cellular heterogeneity, gene regulatory networks, cell-cell interactions, and differentiation trajectories in tissues. This technique has been widely used in oncology, microbiology, neurology, and immunology (20). Yet, it has never been applied to SAIDs research. In this study, we are the first to apply this technique to classic SAIDs, aiming to identify the potential mechanisms of disease flare other than the IL-1 pathway and the response to anti-TNF therapy, and to provide the first transcriptomic resource of SAIDs spectrum diseases.

## 2 Results

### 2.1 Single cell profiling of PBMCs and PMNs in SAID patients

This study enrolled 2 patients with classic SAIDs. One patient was a 20-year-old Chinese woman diagnosed as CAPS, with *NLRP3* D303G variation, and another patient was a 16-year-old Chinese man diagnosed as TRAPS, with *TNFRSF1A* V202D variation. Detailed information can be found in **Supplemental Data S1**. Both patients had high disease activity during the first visit (pre-treatment). The CAPS patient presented urticarial rash, arthritis (interphalangeal joint and knees), lymphadenopathy, hearing loss, and vision loss, while the TRAPS patient was in the intermittent phase of disease episodes and did not show significant symptoms. Both patients received Etanercept treatment, and during the 3-month follow-up visit (on-treatment), both patients reported significant remission of clinical symptoms and no disease relapse.

**Figure 1:**
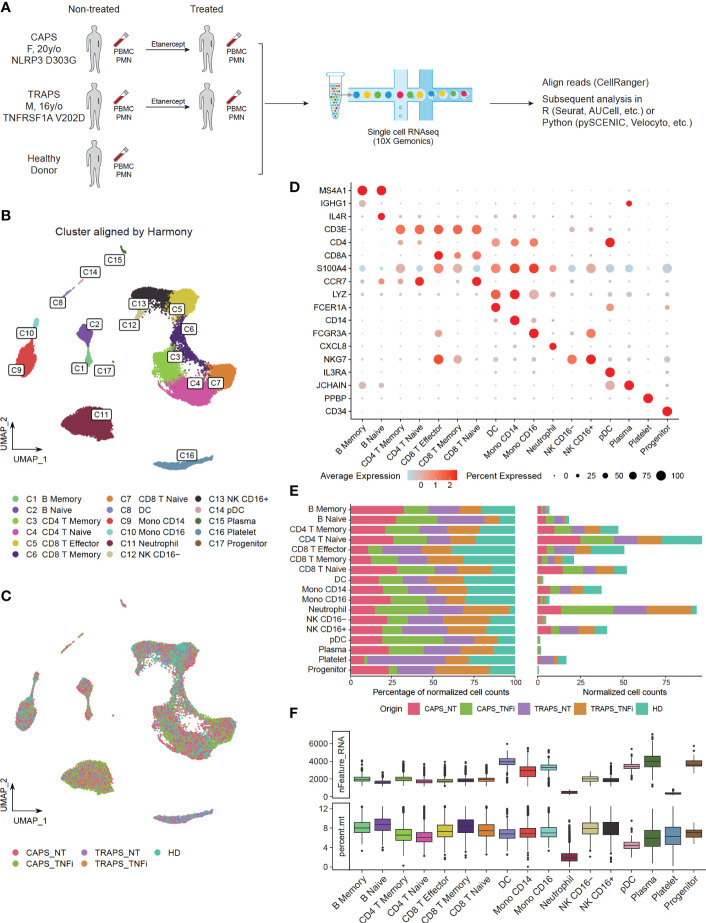
Single Cell Profiling of PBMCs and PMNs in SAID Patients. **(A)** Study design, comparing transcriptomics of PBMCs and PMNs from pre-treatment (CAPS_NT, TRAPS_NT) and Etanercept treated (CAPS_TNFi, TRAPS_TNFi) blood samples of both patients and one healthy donor (HD). **(B, C)** UMAP plot, colored by 17 subtypes **(B)** identified by unsupervised clustering or 5 sample origins **(C)**. Batch effect removed by Harmony algorithm. **(D)** Expression of representative markers of each cell type (y-axis) in 17 clusters (x-axis). Dot size represents the percentage of cells in which the gene is detected. Color indicates the centered mean expression. **(E)** Fraction of 5 sample origins in 17 cell types. Total cell count of each sample was normalized to 100. **(F)** Boxplot displaying the detected gene number (nFeature_RNA) and percentage of mitochondria genes (percent.mt) in each cell type.

Peripheral blood mononuclear cells (PBMCs) and polymorphonuclear neutrophils (PMNs) were extracted from pre-treatment (CAPS_NT, TRAPS_NT) and on-treatment (CAPS_TNFi, TRAPS_TNFi) blood samples of both patients, as well as the blood of one healthy donor (HD). Then the cells were subjected to 3’-scRNAseq (10X Genomics) to study the transcriptomic profiles (**Figure 1A**). As the average detected genes (N_gene_) in different cell types varied dramatically (e.g. neutrophils and platelets had very low N_gene_, while monocytes and plasma cells had higher N_gene_, **Figure 1F**), we used different quality filtering criteria for each cluster (see Materials and methods) and acquired 30,377 cells for the downstream analysis. To identify the common clusters of all 5 samples, Harmony algorithm (21) was used to remove the batch effect, and identified 17 clusters by unsupervised clustering (**Figures 1B, C**), including two B-cell clusters (memory, naïve), plasma cells, five T-cell clusters (memory CD4, naïve CD4, memory CD8, naïve CD8, effector CD8), two NK-cell subtypes (CD16^+^ and CD16^-^), two monocyte subtypes (CD14^+^, CD16^+^), DCs, pDCs, neutrophils, platelets, and progenitors (**Figure 1B**). Representative markers of each cell type were shown in **Figure 1D**. T-cells, CD14 monocytes, CD16 NK-cells, and neutrophils were the more frequent cell types in the blood (**Figure 1E**).

### 2.2 Gene expression profile alters significantly after anti-TNF therapy in CAPS patient

**Figure 2:**
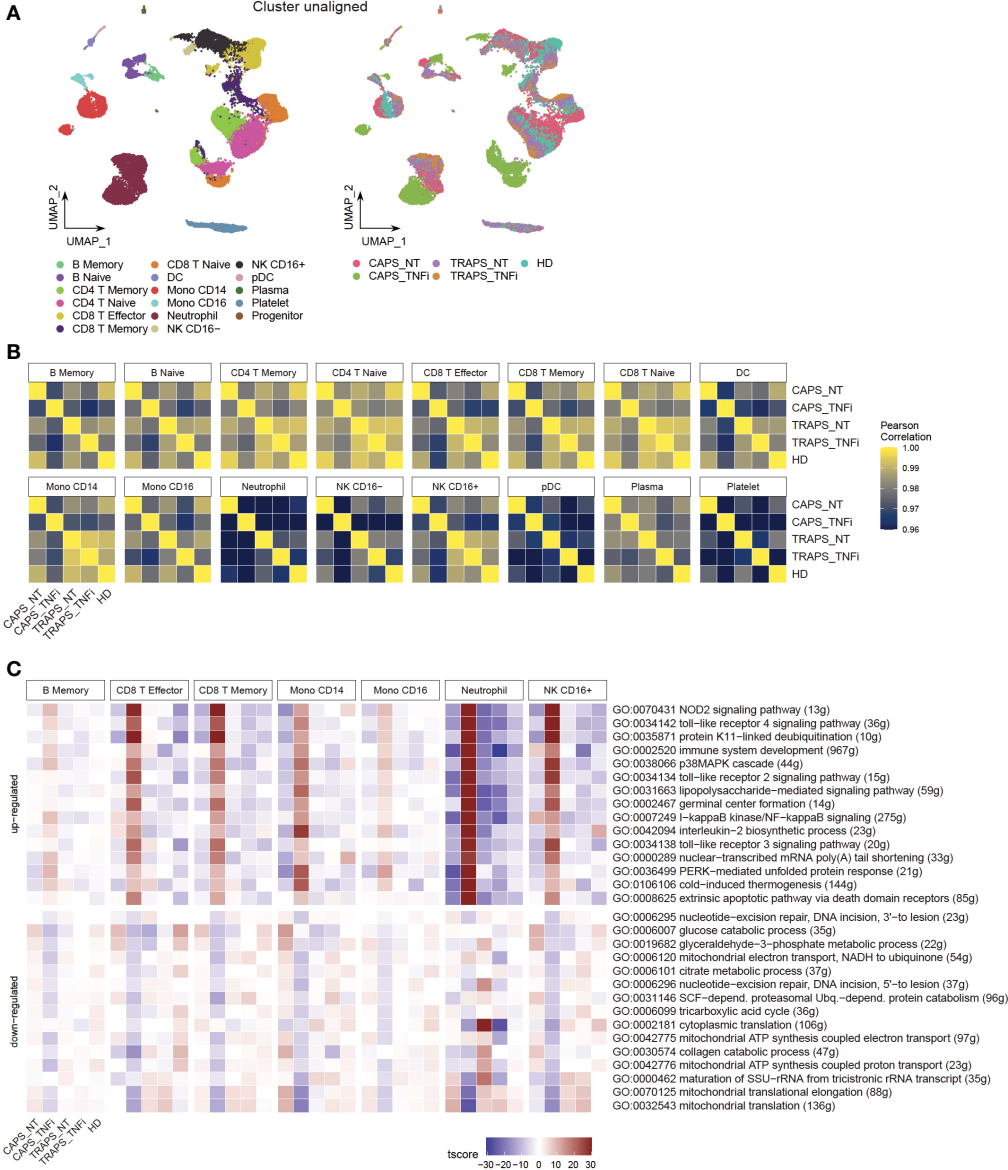
Gene Expression Profile Alters Significantly after Anti-TNF Therapy in CAPS Patient. **(A)** UMAP plot, colored by 17 subtypes (left) or 5 sample origins (right). Batch effect not corrected. **(B)** Pearson correlation of all detected gene expression between samples in each cell type. **(C)** Heatmap showing top 10 up- and down-regulated GO pathways in CAPS_TNFi.

After identifying common clusters, we investigated the differences among these samples in each cell type. Unaligned uniform manifold approximation and projection (UMAP) was plotted based on PCA without Harmony integration and it intuitively showed marked changes in the gene expression profile of CAPS after treatment, compared to other samples (**Figure 2A**). This may suggest that the transcriptome is more associated with disease attack rather than disease activity, as both patients had high activity but only the CAPS patient was at disease flare. To make the comparison more quantitative, we calculated the Pearson Correlation of average gene expression of each cluster between groups (**Figure 2B**). Note that low N_gene_ and cell counts can usually generate lower Pearson Correlation value due to a higher variance, like neutrophils, platelets, DCs/pDCs and CD16^-^ NK cells. In general, CAPS_TNFi shows a lower correlation value compared to other samples, which confirmed our observation in **Figure 2A**. B cells, effector and memory CD8 T cells, NK cells and monocytes had relatively higher variance than naïve or CD4 T cells, indicating that these can be the main cell types that respond to anti-TNF therapy in the CAPS patient.

**Figure 3:**
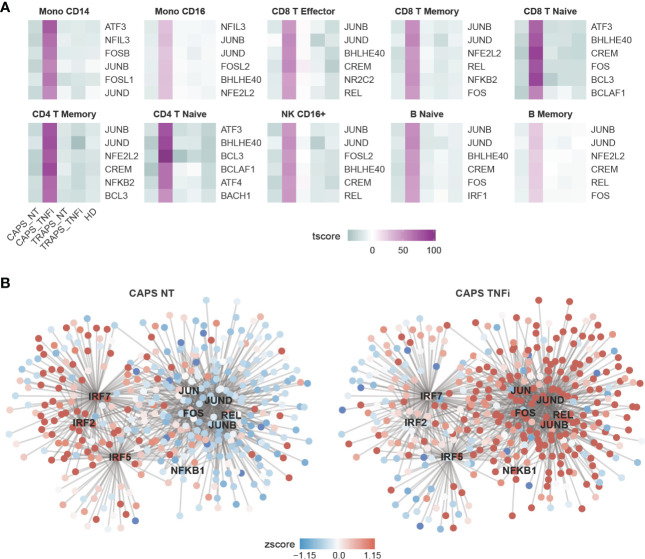
Gene Regulatory Network (GRN) Predicted by SCENIC Analysis. **(A)** Heatmap showing top 6 most activated TFs in CAPS_TNFi predicted by SCENIC. **(B)** Gene regulatory network (GRN) of CD16^+^ monocytes, predicted by SCENIC. The gene expression (round nodes) or regulon activity (square node) in CAPS_NT (left) or CAPS_TNFi (right) is shown as node color (z-score).

To further look into which pathways contribute to the major changes in CAPS_TNFi, Gene Set Enrichment Analysis (GSEA) was performed based on Gene Ontology (GO) database, and identified common top 10 up- and down-regulated pathways in some representative cell types (**Figure 2C**). Surprisingly, many up-regulated pathways after anti-TNF therapy in CAPS were related to stress response and innate immune response-activating signal transduction, including pattern recognition receptor (PRR) signaling pathways like toll−like receptor (TLR) and NOD2 signaling pathways. Meanwhile, some metabolic pathways related to aerobic respiration were down-regulated (**Figure 2C**). To confirm this, SCENIC analysis (22) was performed which inferred the gene regulatory network and predicted regulon (transcription factor, TF) activities, and found that indeed many inflammatory-related TFs are activated in CAPS_TNFi, including canonical NFκB pathway TFs (NFKB1, REL) and AP-1 complex (FOS, JUN) (23) (**Figures 3A, B**). We reasoned that the upregulation of inflammatory pathways could be a compensatory adjustment to anti-TNF therapy, although this requires further validation.

Although gene expression changes were less pronounced in the TRAPS patient after anti-TNF treatment than in CAPS, we also performed a comprehensive analysis of differentially expressed genes (DEGs), up/downregulated TF activities and GO pathways in the TRAPS patient, which are documented in **Supplementary Data S2**. The same analysis was performed comparing CAPS_NT/TRAPS_NT to healthy donors to identify the potential pathogenic genes in SAID (**Supplementary Data S2**). However, this may be difficult due to the small sample size, as some DEGs may be individual-specific (e.g. genes on the Y chromosome, ribosomal proteins) and apparently not associated with the disease. These data may be more useful when more SAID data becomes available in the future.

### 2.3 Disease flare is unnecessary to be positively correlated with serum inflammatory cytokines

**Figure 4:**
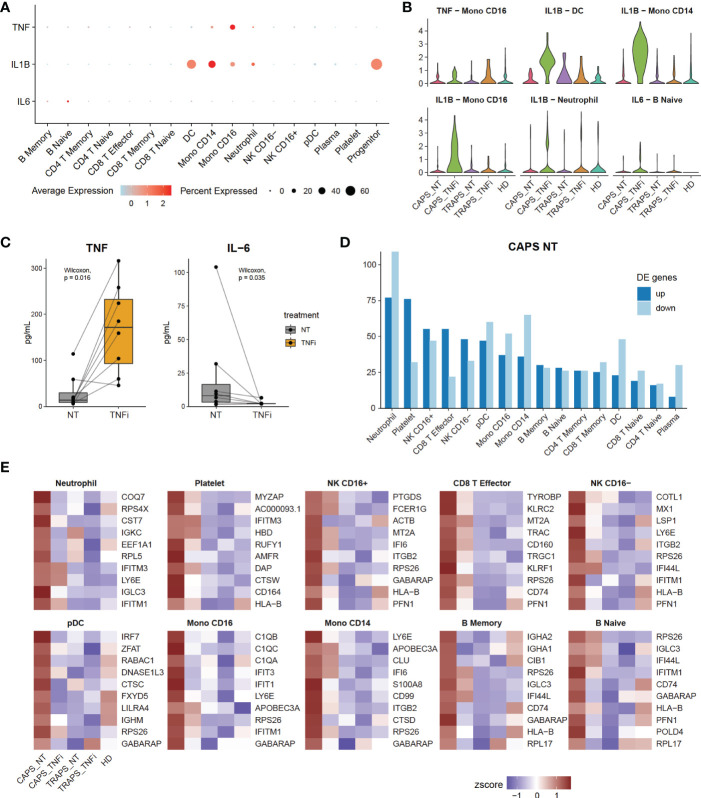
Disease Flare Is Unnecessary to Be Positively Correlated with Inflammatory Cytokines. **(A)** Expression of inflammatory markers TNF, IL-1 and IL-6 of each cell type (y-axis) in 17 clusters (x-axis). Dot size represents the percentage of cells in which the gene is detected. Color indicates the centered mean expression. **(B)** Violin plots displaying the expression of inflammatory markers in their main source cell types, comparing 5 sample origins. **(C)** Serum TNF and IL-6 levels in SAID patients, comparing before and after anti-TNF therapy. **(D)** Bar plot showing the number of up- and down-regulated differentially expressed genes (DEGs) of CAPS_NT of each cell type. **(E)** Heatmap showing top 10 up-regulated DEGs in CAPS_NT.

One important clinical question is which markers are elevated during disease attack. Normally, we evaluate clinical manifestations and laboratory tests including white blood cell (WBC) count, erythrocyte sedimentation rate (ESR), and C-reactive protein (CRP), etc (24). Some inflammatory cytokines, like TNF, IL-1β and IL-6 are considered to be the cause of systemic inflammation, although IL-6 has both pro- and anti-inflammatory functions (25). Thus, we asked how these inflammatory cytokines changed after anti-TNF therapy. First, we checked which cell types were the main source of each cytokine (**Figure 4A**), and found that TNF was mainly secreted by CD16^+^ monocytes, IL-1β mainly by monocytes, DCs and neutrophils, and IL-6 by a small subset of B cells. Next, the expression levels of these cytokines were examined in the relevant clusters. We noticed that in general they were even more highly expressed after anti-TNF treatment (**Figure 4B**). This appears counterintuitive, while similar to what we found in **Figure 2C**. Thus, we asked if the increase of inflammatory cytokines after anti-TNF treatment occurred commonly, by chance, or was just a technical effect of scRNAseq. Therefore, we tried to validate this using other methods. As some patients were tested for serum TNF and IL-6 during their visit to the hospital, we checked if these cytokines were increased after anti-TNF treatment at the protein level. We reviewed our SAIDs cohort from 2015 (26), until Jan 2021, and among total of 228 patients, 34 were received anti-TNF therapy (either Etanercept, Infliximab, Golimumab or Adalimumab), and 8 patients were tested for TNF and IL-6 both before and after anti-TNF therapy. The serum TNF levels were increased while IL-6 decreased after the treatment (**Figure 4C**). Although it is still not clear what role IL-6 plays during the treatment, we confirmed that TNF was increased after anti-TNF therapy, by both scRNAseq and serum protein test. Taken together, the inflammatory cytokines, at least TNF and IL-1β can be up-regulated after anti-TNF therapy and are not necessarily positively correlated with disease flare.

Next, we asked what changes reflect the disease attack, if not TNF or IL-1β. As we know that among the 5 samples, the CAPS_NT sample was taken during disease attack while other samples were not. Therefore, the DEGs of CAPS_NT in each cell type were checked (**Figures 4D, E**). We noted that some interferon-induced genes, including IFI, IFIT and IFITM families, which are involved in broad-spectrum antiviral functions (27) were upregulated in CAPS_NT, remarkably in the CD16^+^ monocyte subtype (**Figure 4E**). Consistently, by SCENIC analysis we also observed that several interferon regulatory factors, e.g. IRF2, 5 and 7 were activated in the CAPS_NT sample in CD16 monocytes (**Figure 3B**). These data indicated that the interferon response might play a role in the disease flare.

We further investigated whether the increased interferon response in CAPS_NT was caused by elevated stimuli levels (IFN-α, IFN-γ), or enhanced sensitivity in these monocytes. First, IFN-α and IFN-γ were checked at both RNA and protein levels. In the single-cell RNA-seq data, we did not see increased IFN expression in non-treated samples (**Figure S1A**). At the protein level, we acquired several serum samples of non-treated SAID patients (CAPS = 4, TRAPS = 1) from the biobank in our hospital as well as 3 healthy donors, and tested IFN-α and IFN-γ levels by ELISA. Consistent with scRNAseq data, there was no increased serum IFN in non-treated SAID samples (**Figure S1B**). We then checked the IFN signaling pathways in CD16 monocytes. The IFN signaling gene lists from the Reactome database (28) were used and calculated the enrichment score using the AUCell package (22). We found that indeed there was higher activation of IFN signaling pathways in CAPS_NT (**Figure S1C**). These data supported that the increased interferon response in CAPS_NT was probably caused by enhanced sensitivity to the IFN stimuli in CD16 monocytes, rather than elevated ligand levels.

### 2.4 Inhibition of macrophage differentiation is a potential mechanism of anti-TNF therapy

**Figure 5:**
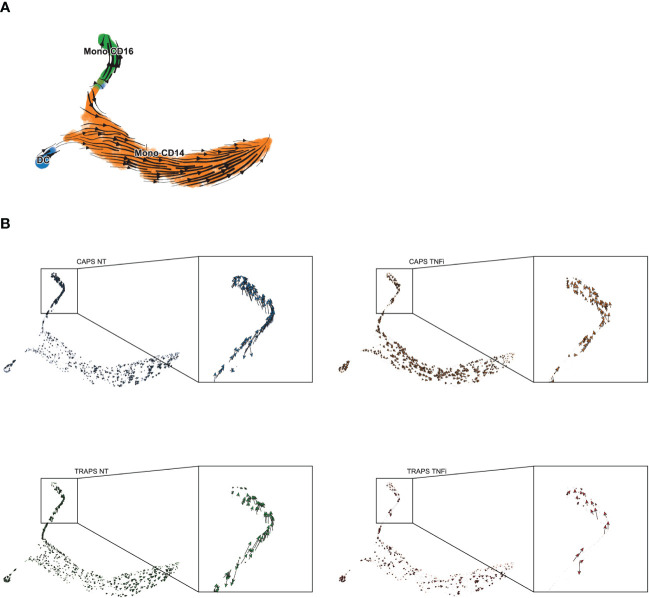
Differentiation Trajectory Analysis of Monocytes. **(A, B)** Differentiation trajectory of NT/TNFi monocytes, predicted by Velocyto/ScVelo, based on tSNE embeddings calculated by Palantir. Differentiation direction shown by arrows, in all samples **(A)** or split by each sample **(B)**.

From the previous analysis, we also noticed that complement components (C1Qs) were more upregulated in pre-treatment than on-treatment samples in CD16^+^ monocytes (**Figure 4E**), which is one of the key features of macrophages (29). Interestingly, CD16^+^ monocytes indeed resemble macrophages in many aspects, as they have low expression of classic monocyte markers like CD14 and CCR2, and highly express CX3CR1 which explains why they migrate and adhere more than CD16^−^ monocytes to fractalkine-secreting endothelium, and specialize in complement and FcR-mediated phagocytosis and anti-viral responses (30–32). The lower expression in complement after TNF blockade gave us a hint that the treatment might change the differentiation state of CD16^+^ monocytes. To validate this hypothesis, pseudo-time trajectory analysis was performed within monocyte clusters using Palantir and scVelo/Velocyto (33–35) which could predict the direction of differentiation and “RNA velocity” — the time derivative of the gene expression state — based on the relative abundance of nascent (unspliced) and mature (spliced) mRNA (**Figure 5A, B**). We found that before treatment, the CD16^+^ monocytes were highly dynamic and differentiating into the terminal state, while after TNF blocking, the differentiation was blocked, depicted by the arrows with shorter lengths than untreated (**Figure 5B**). Indeed, TNF is known to be involved in macrophage differentiation (36). This analysis shows that inhibition of macrophage differentiation, rather than reducing the overall level of inflammatory cytokines, could be a potential mechanism of anti-TNF treatment response in SAIDs.

## 3 Discussion

In this study, we for the first time described the transcriptomic profile of PBMCs and PMNs in SAIDs patients, and also revealed transcriptional changes after anti-TNF therapy. We observed that after anti-TNF therapy, some pathways related to stress response and innate immune response-activating signal transduction were up-regulated, while some metabolic pathways related to aerobic respiration were down-regulated in CAPS, whereas there were no significant changes in TRAPS. This may suggest that the transcriptome is more associated with disease attack rather than disease activity, and may partially explain the unexpected good response to IL-1 blockade compared with anti-TNF therapy observed in TRAPS patients in previous studies (37, 38). We also identified that inflammatory cytokines such as TNF and IL-1β were up-regulated after anti-TNF therapy and were not necessarily positively correlated with disease activity. Interestingly, some interferon-induced genes were up-regulated in the CAPS pre-treatment sample, which indicated that the interferon response can be correlated with disease flare. Further analysis revealed that one of the potential mechanisms of anti-TNF treatment response in SAIDs may be the inhibition of macrophage differentiation rather than decreasing the overall level of inflammatory cytokines. As macrophages are a major cell type involved in inflammation, and their activation can be uncontrolled in SAIDs due to the genetic defect, inhibition of macrophage differentiation may help reduce the frequency of disease flare.

As SAIDs are rare diseases, in this exploratory study where we only enrolled 2 patients (one CAPS and one TRAPS) to test the feasibility of using scRNAseq for SAIDs research. As the number of samples was limited, we could not gain robust conclusions from this study. However, we can see that after anti-TNF therapy, we observed significant transcriptional changes after treatment in CAPS patient, indicating that scRNAseq can be a useful technique for the future study of SAIDs and treatment response. Meanwhile, we are also aware of the shortcomings of this study design, which can be improved in the future: i) Some differentiated immune cells in the peripheral tissues (e.g. myeloid cells in skin lesions or synovial fluid) may have important pro-inflammatory functions. Therefore, we should consider not only extracting circulating PBMCs/PMNs but also collecting samples from peripheral tissues. ii) Some long-term effects caused by genetic variation or treatment may be the result of epigenetic modifications that cannot be revealed by scRNAseq, which is more reflective of immediate changes in cell state. Novel techniques such as single cell assay for transposase-accessible chromatin (ATAC) together with RNA sequencing may help us better understand such mechanisms. iii) In the study design we included neutrophils as they have important pro-inflammatory functions. However, we then realized that they had very few transcripts detected and less usable sequencing reads, as they had relatively low RNA content and relatively high levels of RNases with other inhibitory compounds. This brought great challenges to the subsequent analysis as we could not get high-quality information from these cells. In future studies, we should consider either supplementing RNase inhibitor to preserve the RNA or only keeping other high-quality cells without neutrophils.

In conclusion, these results may indicate that interferon response, rather than TNF or IL-1, may be involved in the disease flare of the SAIDs. Trajectory analysis showed that inhibition of macrophage differentiation as the potential mechanism of anti-TNF treatment response in SAIDs. Our work provided new insights into the potential mechanisms of disease flare and anti-TNF treatment response, new transcriptomic resources of SAID spectrum diseases, and expanded the application of single-cell RNA sequencing technology to new disease areas.

### 3.1 Limitations of the study

As an exploratory study, the main limitation of this study is the small sample size, making it impossible to generalize in this situation. In addition, there is a lack of appropriate mouse models to validate our conclusions. The aim of the further research should be to expand the study cohort and apply multi-omics technology (e.g. single-cell RNA-seq, scATAC-seq and sc-proteomics) to various tissue types (e.g. PBMC, skin lesion or synovial fluid) to gain further insight into the pathogenesis of SAIDs, and to validate the findings by genetically engineered mouse models (GEMM) that mimic the clinical phenotypes.

## 4 Materials and methods

### 4.1 Patients

This study enrolled 2 patients with classic SAIDs. One patient was a 20-year-old Chinese woman diagnosed as CAPS, with *NLRP3* D303G variation, and another patient was a 16-year-old Chinese man diagnosed as TRAPS, with *TNFRSF1A* V202D variation. Detailed information can be found in **Supplemental Data S1**. Both patients were in the cohort of adult SAID patients in Peking Union Medical College Hospital (PUMCH), which was described in the previous publication (26). This study was approved by the Institutional Review Board of Peking Union Medical College Hospital and performed according to the Declaration of Helsinki. Informed consents were obtained from all participants.

### 4.2 Single-cell RNA sequencing

#### 4.2.1 Sample preparation

The processing of blood samples, including PBMC and neutrophil isolation, library preparation, and single-cell RNA sequencing were done by CapitalBio Technology, Beijing. In brief, ACCUSPIN™ System-Histopaque^®^-1077 and Histopaque^®^-1119 reagent were used to isolate mononuclear cells and granulocytes, respectively, following the protocol on Sigma-Aldrich website. Using single cell 3’ Library and Gel Bead Kit V3 (10x Genomics, 1000075) and Chromium Single Cell B Chip Kit (10x Genomics, 1000074), the cell suspension (300-600 living cells per microliter determined by Count Star) was loaded onto the Chromium single cell controller (10x Genomics) to generate single-cell gel beads in the emulsion according to the manufacturer’s protocol. In short, single cells were suspended in PBS containing 0.04% BSA. About 6,000 cells were added to each channel, and the target cell will be recovered was estimated to be about 3,000 cells. Captured cells were lysed and the released RNA were barcoded through reverse transcription in individual GEMs. Reverse transcription was performed on a S1000TM Touch Thermal Cycler (Bio Rad) at 53°C for 45 min, followed by 85°C for 5 min, and hold at 4°C. The cDNA was generated and then amplified, and quality assessed using an Agilent 4200 (performed by CapitalBio Technology, Beijing).

#### 4.2.2 Data processing and cell clustering

Raw sequencing data (fastq files) were mapped to the human genome (build GRCh38) using CellRanger software (10x Genomics, version 3.0.2). Raw gene expression matrices generated per sample were analyzed with the Seurat package in R (39). To achieve clean cell clustering results, we divided the cell filtering process into two major steps: *primary clustering* and *fine adjustment*. *Primary clustering*: 5 samples (CAPS_NT, CAPS_TNFi, TRAPS_NT, TRAPS_TNFi, HD) were merged together and cells were filtered by nFeature_RNA (genes detected) > 200 and percent.mt (percentage of mitochondria genes) < 12.5%. High variable genes were selected by FindVariableFeatures and auto-scaled by ScaleData function using default parameters, and a principal component analysis (PCA) was performed for all datasets using the default RunPCA function in the Seurat package. Batch effect correction of each sample was done using the Harmony algorithm (21) based on PCA space, followed by FindNeighbors and FindClusters function (dims = 30, resolution = 0.5) in the Seurat package for unsupervised clustering. In total 20 clusters were found. As plasma cells were not correctly identified by unsupervised clustering, they were manually annotated. *Fine adjustment*: scDblFinder package was used to predict potential doublets in the datasets (40). As neutrophils and platelets naturally have much fewer transcripts than other cell types, and DCs are often misclassified as doublets, we divided all cell types into 3 groups and use different filter criteria for each group. Group1: including Naïve CD4, Naïve CD8, Memory CD4, Memory CD8, Effector CD8, CD16^+^ NK, CD16^-^ NK, CD14 Mono, Naïve B, and Memory B, these cells were filtered by nFeature_RNA > 1200 and kept only singlets by scDblFinder; Group 2: including Neutrophil and Platelet, these cells were filtered by nFeature_RNA < 800 and kept only singlets by scDblFinder; Group 3, including FCGR3A Mono, DC, and pDC, these cells were filtered only by nFeature_RNA > 1200 regardless of scDblFinder prediction. In addition, 1 RBC cluster, 3 doublet clusters and 1 mitotic cluster were removed as they were not informative. All plasma cells were kept manually. After filtering, we ran the same pipeline as primary clustering mentioned above with slightly changes of several parameter (dims = 20, resolution = 0.4). Final clustering results were shown in **Figure 1B**.

#### 4.2.3 Differential expression analysis and data visualization

To visualize cells on a 2D plot, Uniform manifold approximation and projection (UMAP) was done by RunUMAP function (dims = 20) in Seurat. Differentially expressed genes (DEGs) were identified by the Wilcoxon Rank Sum test using the FindMarkers function. Gene expression levels or gene set enrichment scores (AUCell score) were shown in t-score or z-score for heatmaps or waterfall plots. UMAP or tSNE plots were done using DimPlot or FeaturePlot functions in Seurat. Heatmaps, modified stacked violin plots, boxplots, and bar plots of cluster proportion were generated using customized codes in R, and these functions were integrated into the R package “SeuratExtend” which is available on Github (https://github.com/huayc09/SeuratExtend).

#### 4.2.4 SCENIC and gene set enrichment analysis

To carry out the transcription factor network inference, SCENIC workflow was performed using Nextflow pipeline (22), and regulon activity of each cell was evaluated using AUCell score with Bioconductor package AUCell. For functional/pathway analysis, gene set lists were collected from databases including Gene Ontology (GO) and Reactome. For Gene Set Enrichment Analysis (GSEA), the enrichment of given gene sets of each cell was evaluated using AUCell package as well. Gene regulatory networks (**Figure 3**) were plotted using Cytoscape software (41).

#### 4.2.5 Trajectory analysis

For trajectory analysis (**Figure 5**), monocyte subsets of CAPS and TRAPS samples were extracted and integrated by Harmony algorithm. Python package Palantir (34) was used to calculate diffusion map and diffusion components based on Harmony space, then cells were visualized by tSNE. To predict the differentiation direction, we conducted a Velocyto pipeline (33) using the *.bam file and barcode information generated by CellRanger, and used ScVelo in Python (35) for better visualization.

### 4.3 ELISA

The plasma levels of IFN-α and IFN-γ were detected according to the instructions of the commercial ELISA kits (EXCELL Bio, China).

### 4.4 Statistical analysis

Differentially expressed genes (DEGs) were identified by the Wilcoxon Rank Sum test using the FindMarkers function of Seurat package in R. All remaining statistical analysis were performed by the ggpubr package in R with default parameters.

## Data availability statement

The raw sequence data reported in this paper have been deposited in the Genome Sequence Archive (42) in National Genomics Data Center (43), China National Center for Bioinformation / Beijing Institute of Genomics, Chinese Academy of Sciences (GSA-Human: HRA003572) that are publicly accessible at https://ngdc.cncb.ac.cn/gsa-human. All software is freely or commercially available. To ensure data accessibility to non-bioinformaticians, we made the an interactive web tool generated by ShinyCell (44) at https://yichao-hua.shinyapps.io/shen_lab_said_anti-tnfa/.

## Ethics statement

The studies involving human participants were reviewed and approved by Institutional Review Board of Peking Union Medical College Hospital. Written informed consent to participate in this study was provided by the participants’ legal guardian/next of kin. Written informed consent was obtained from the individual(s), and minor(s)’ legal guardian/next of kin, for the publication of any potentially identifiable images or data included in this article.

## Author contributions

MS and YH conceived the study. YH performed the bioinformatics analyses. NW performed the Elisa experiment. JM summarized the medical history of SAID cohort. YH and MS wrote the manuscript. All authors contributed to the article and approved the submitted version.

## Supplementary material

Supplementary Data Sheet 2 Differentially expressed genes, regulon activities and GSEA
