# Supplementary material for: Single-cell transcriptomic analysis in two patients with rare systemic autoinflammatory diseases treated with anti-TNF therapy
Source: Front Immunol. 2023 Feb 24;14:1091336. doi: 10.3389/fimmu.2023.1091336 (PMC9998688; doi:10.3389/fimmu.2023.1091336)
Supplement: Supplementary file 3 [file Image_1.pdf]

Supplemental figure 1

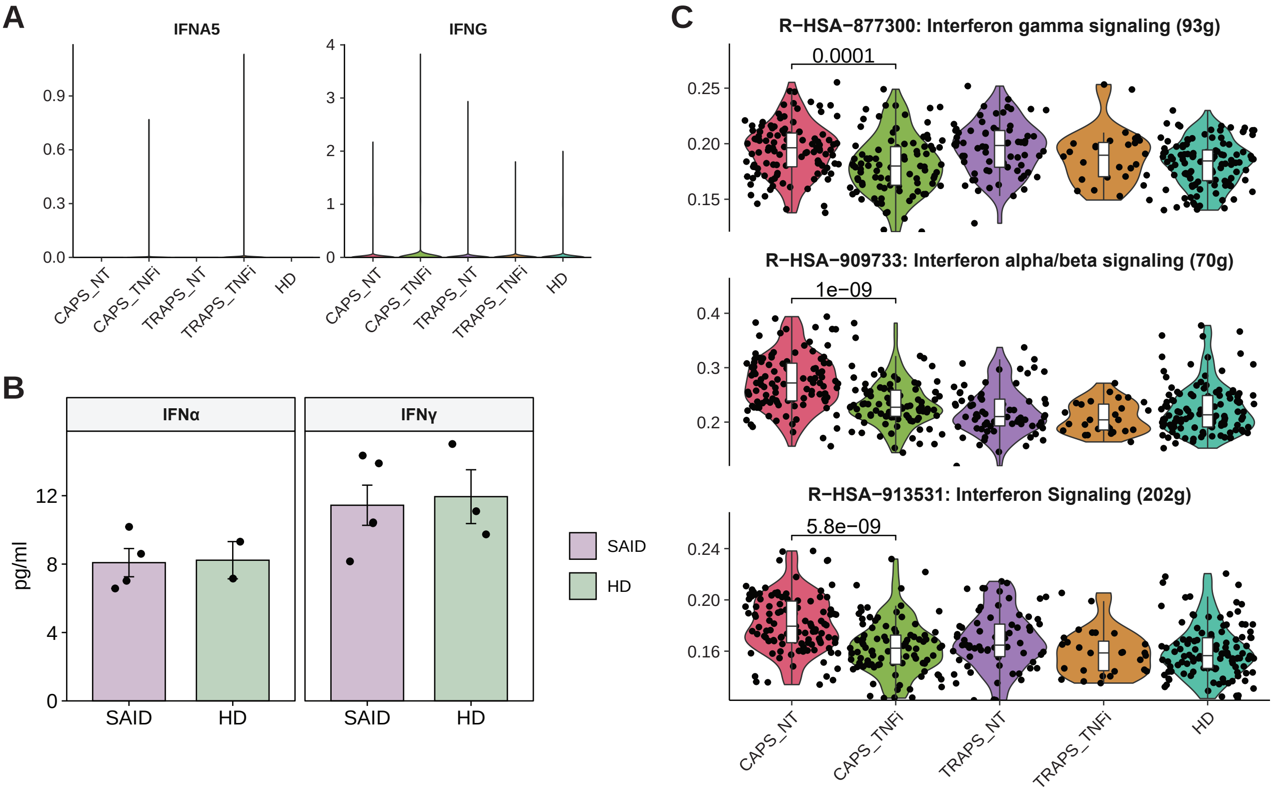

Figure S1. Interferons and Interferon Signaling Pathways.
(A) Violin plots showoing the expression of IFNs in all cells, comparing 5 sample origins.
(B) Serum IFNα and IFNγ levels in non-treated SAID patients and healthy donors by ELISA.
(C) Violin plots showing the AUCell enrichment scores of interferon signaling gene lists from Reactome database in CD16+ monocytes, comparing 5 sample origins.
